# Evaluation of Physical and Functional Protein-Protein Interaction Prediction Methods for Detecting Biological Pathways

**DOI:** 10.1371/journal.pone.0054325

**Published:** 2013-01-17

**Authors:** Vijaykumar Yogesh Muley, Akash Ranjan

**Affiliations:** 1 Computational and Functional Genomics Group, Centre for DNA Fingerprinting and Diagnostics, Hyderabad, India; The Centre for Research and Technology, Hellas, Greece

## Abstract

**Background:**

Cellular activities are governed by the physical and the functional interactions among several proteins involved in various biological pathways. With the availability of sequenced genomes and high-throughput experimental data one can identify genome-wide protein-protein interactions using various computational techniques. Comparative assessments of these techniques in predicting protein interactions have been frequently reported in the literature but not their ability to elucidate a particular biological pathway.

**Methods:**

Towards the goal of understanding the prediction capabilities of interactions among the specific biological pathway proteins, we report the analyses of 14 biological pathways of *Escherichia coli* catalogued in KEGG database using five protein-protein functional linkage prediction methods. These methods are phylogenetic profiling, gene neighborhood, co-presence of orthologous genes in the same gene clusters, a mirrortree variant, and expression similarity.

**Conclusions:**

Our results reveal that the prediction of metabolic pathway protein interactions continues to be a challenging task for all methods which possibly reflect flexible/independent evolutionary histories of these proteins. These methods have predicted functional associations of proteins involved in amino acids, nucleotide, glycans and vitamins & co-factors pathways slightly better than the random performance on carbohydrate, lipid and energy metabolism. We also make similar observations for interactions involved among the environmental information processing proteins. On the contrary, genetic information processing or specialized processes such as motility related protein-protein linkages that occur in the subset of organisms are predicted with comparable accuracy. Metabolic pathways are best predicted by using neighborhood of orthologous genes whereas phyletic pattern is good enough to reconstruct central dogma pathway protein interactions. We have also shown that the effective use of a particular prediction method depends on the pathway under investigation. In case one is not focused on specific pathway, gene expression similarity method is the best option.

## Introduction

Proteins are responsible for almost every cellular function of an organism such as behavior, metabolic activities and other phenotypic traits. Since proteins never work in isolation, identifying cellular function is crucial for understanding their role at system level. The cellular function of a protein is equivalent to the biological process/pathway in which it participates [1]. Cellular function prediction efforts are accelerated with the availability of completely sequenced genomes of several organisms and high-throughput experimental techniques [1], [2], [3], [4], [5], [6], [7]. Although genomes rearrange dynamically in the course of evolution, it was observed that the functional or regulatory contexts of the proteins are invariably maintained [8], [9], [10], [11], [12]. Hence, it is possible to predict cellular function or context of a protein based on the analysis of evolutionary aspects shared with other cellular proteins using genomic sequences [6], [13], [14]. One assumption seeks that concerted appearance or disappearance of proteins in various organisms, which is likely due to the same functional constraints operational on them [12], is referred as Phylogenetic Profiling (PP) [15]. Chromosomal proximity of genes reflects their co-regulation under the same functional constraints [11], [16]. However, prokaryotic genomes are often rearranged randomly that breaks down genomic neighborhood of genes even in closely related species [9]. Given a genome, it is possible to identify such rearranged genes and possible functional linkages between their products based on the chromosomal proximity of genes encoding their orthologs in multiple genomes by using two possible approaches. Gene Neighbor (GN) method which identifies rearranged genes based on the genomic neighborhood of the orthologous genes independent of directionality (gene order) while Gene Cluster (GC) considers co-directional proximity of orthologous genes [17], [18], [19], [20], [21], [22]. There is also another class of methods, called as mirrortree type method, based on the similarity of phylogenetic trees of two interacting protein families [23], [24], [25]. Functional linkage can also be inferred using Expression Similarity (ES) of genes in various physiological conditions by assuming their requirement at the same time [26], [27].

Reconstruction of biological pathways is possible through genome-wide physical/functional Protein-Protein Interactions (PPIs) predicted by these methods. However, only few studies have addressed the benefits and the limitations of each method to reconstruct biological pathways [4], [28], [29], [30], [31], [32]. Notably, Karimpour-Anis and co-workers showed that the use of gene fusion, GN, GC and PP method depends on tasks by comparing pathway, operon reconstruction and various functional aspects [30]. However, they did not emphasize on the prediction of independent biological pathways by these methods in sufficient details. Jothi and coworkers have done exhaustive pathway prediction analysis but their study was limited to PP method and focused on the effect of reference genome selection on prediction accuracy of methods [29].

Considering the independent evolutionary histories of pathways [29] and the unique features used by these methods [33], comparative analysis to predict individual biological pathway has implications for later uses of these approaches. To this end, we report here comprehensive analyses of these methods in predicting biological pathways of *Escherichia coli* K12 MG1655 (*E. coli*). Our results identify the best method suited for the prediction of biological pathway interactions. These results also provide pointers to the evolutionary and co-regulatory constraints on the proteins.

## Results and Discussion

**Figure 1:**
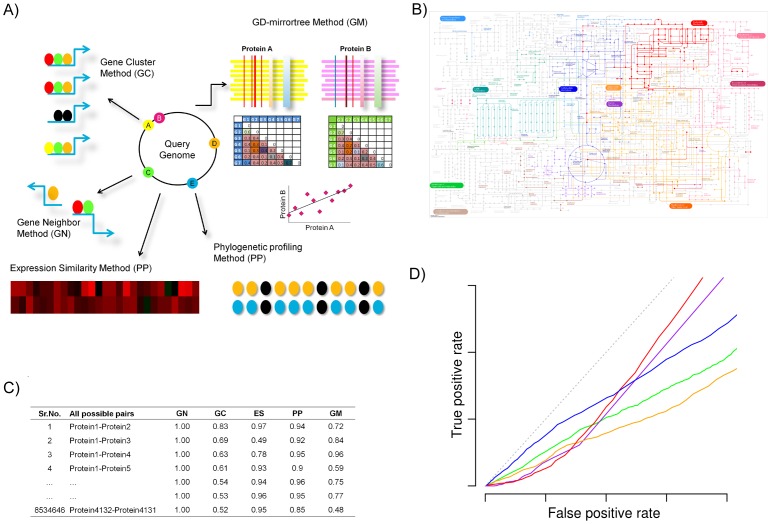
A schematic representation of the approach. A) Each arrow point towards a particular protein-protein physical/functional interaction prediction method. Gene Cluster (GC) calculates co-occurrence probability of orthologs of query proteins encoded from same gene clusters in reference genomes. Gene clusters were defined as a set of unidirectional genes within intergenic distance of 100 nucleotide bases. In given example, genes encoding orthologs of query proteins C and D co-occur in same cluster in three reference genomes, hence 3/4 is interaction score between them. Gene Neighbor (GN) method calculates interaction scores for query protein pairs based on the minimum chromosomal distance between their orthologs encoding genes in any one reference genome irrespective of gene orientation. In given example, minimum distance for proteins C and D evident in one of the reference genome. Expression Similarity (ES) method correlates expression profiles of protein coding genes in various conditions. In given plot, two genes show almost similar expression in various conditions and hence are likely to be interacting. Phylogenetic Profiling (PP) calculates interaction scores based on co-occurrence of proteins in multiple genomes. Phyletic pattern of orthologs of E and D proteins showed with colored filled circles in rows while, vertical stacking represents an individual reference genome. Black circles represents absence of ortholog otherwise presence. Genome distance-Mirrortree (GM) method compares distance matrices derived from aligned orthologs of query proteins. Prior to comparison, we correct these matrices to exclude speciation information using new approach. B) A set of 14 pathways catalogued in KEGG were used as benchmarking dataset. Protein pairs that co-occur in pathway under consideration (for example, Nucleotide metabolism highlighted with red color) were treated as positives and all other pathway protein pairs considered as negatives. C) We calculate interaction scores using above mentioned five methods for positives and negatives of each pathway as shown in table. D) We compare performance accuracy of protein-protein interaction prediction methods for each KEGG pathway using Receiver Operator Characteristics curves.

A schematic representation of five PPI prediction methods used in our analysis and overall approach is given in Figure 1. These methods are GN, GC, ES, PP and a Tol-mirrortree [25] variant called Genome distance-Mirrortree (GM) [34]. The description of each method is given in Text S1 and computational cost in Table S1. We evaluated 969,528 pairs among 1,393 *E. coli* proteins for which pathway memberships were recorded in the Kyoto Encyclopedia of Genes and Genomes (KEGG) database [35]. This dataset included 53,424 positive pairs among proteins that share at least one KEGG pathway at 3rd level of KEGG Orthology (KO) definition. These positive pairs were considered as functionally linked and constituted our positive gold standard whereas remaining pairs treated as negatives. Each method generates numerical values or interaction scores for pairs of proteins on 0 to 1 scale. Except GN method, the scores generated towards 1 tend to represent strong evidence of functional linkage between two proteins whereas 0 represents no functional linkage. The scores generated by GN were normalized distances (on scale 0–1) between genes encoding two proteins on chromosome of reference genome. These distances were subtracted from one to make them interaction (likelihood) scores.

### General Observations

**Figure 2:**
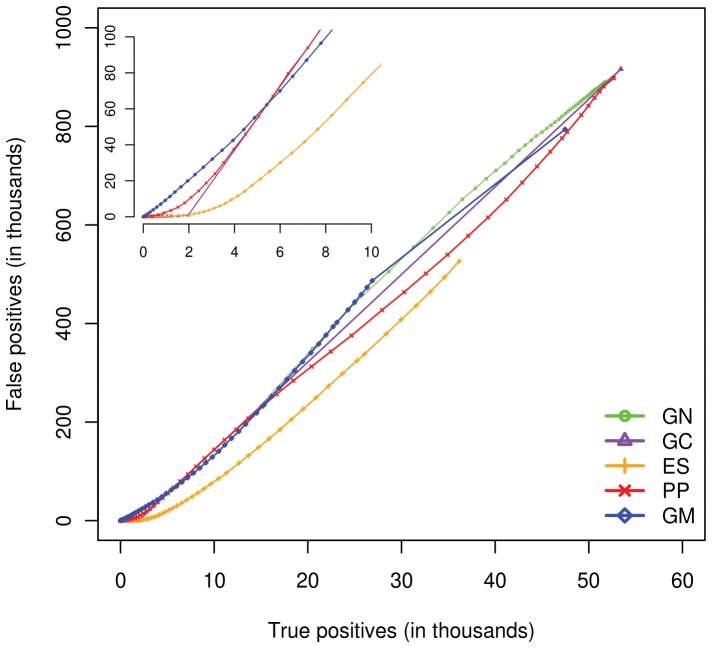
Predictive power of physical and functional protein-protein interaction prediction methods. Each point on this plot represents specific interaction score threshold of prediction methods at which the true and false positives were counted. Inset on the plot shows reduction of false positives at higher interaction score cutoffs for GC, PP, ES and GM prediction methods. The performance of all methods is near diagonal. Even at highest score cutoff GN predicted more than 13,868 TP and 210,963 FP hence its line is not visible in the inset. Expression Similarity (ES) is the best performing method. Phylogenetic Profiling, Gene Neighbor, Gene Cluster and Genome Distance-Mirrortree are abbreviated as PP, GN, GC and GM respectively.

True Positives (TP) and False Positives (FP) were recorded at a series of interaction score thresholds generated for KEGG protein pairs by each prediction method. TP were the pairs with the proteins that share at least one KEGG pathway and their scores are equal to or above threshold. If they do not share KEGG pathway they were treated as FP. Figure 2 represents the numbers of FP and TP recorded at series of thresholds scores. Even at high prediction scores of these methods, around 10 thousand TP were detected at the cost of more than 100 thousand FP suggesting 9 incorrectly identified pairs for each true prediction (Figure 2 inset). ES method was the best performer in predicting functional linkages among proteins reported in the KEGG database. Our finding is consistent with the previous study in Yeast, where ES was ranked as the best feature to classify physical, functional and co-complex PPIs [31]. The second best performer was PP while GN and GM performed relatively poor. TP and FP predicted by GN at its score cutoff of 0.98 or above was around 13 and 210 thousands respectively which suggesting high coverage of true predictions at the cost of higher number of false positives.

The positive examples were further categorized into 14 distinct pathways or functional categories with 50 or more proteins at 2nd level of KO definition, such as nucleotide and lipid metabolism (Table 1). These 14 pathway protein pairs were used as gold standard to plot performance of prediction methods using Receiver Operator Characteristics (ROC) curves. Considering the higher number of FP predicted by these methods, we used ROC as a fair performance measure over others. Here, ROC curves represent the extent with which a particular method ranks individual pathway protein pairs. ROC curves for each pathway prediction using five methods are summarized as the Area Under the ROC Curve (AUC) value in Table 2. For ROC analysis, we considered the protein pair TP, if both the proteins belonged to the pathway under consideration and all other pathway pairs were treated as true negatives (Table 1).

**Table 1:** Description of the KEGG benchmark dataset.

KEGG Pathway	Proteins	Number of Positives	Number of Negatives
All pathways	1393	53424	916104
Amino Acid Metabolism	200	4612	41421
Carbohydrate Metabolism	304	8266	37767
Nucleotide Metabolism	112	4993	41040
Lipid Metabolism	70	1262	44771
Metabolism of Cofactors & Vitamins	145	1974	44059
Energy Metabolism	135	3756	42277
Glycan Biosynthesis & Metabolism	54	536	45497
Metabolism of Other Amino Acids	67	1509	44524
Signal Transduction	129	8690	37343
Membrane Transport	253	17437	28596
Cell Motility	51	1962	44071
Replication and Repair	56	1222	44811
Folding, Sorting and Degradation	52	297	45736
Translation	191	2196	43837

*E. coli* pathways are based on the 2^nd^ level KEGG orthology definition with 50 or more protein components only. Positive pairs are with proteins that share at least one KEGG pathway at the 3^rd^ level of KEGG orthology definition.

**Table 2:** Summary of biological pathway prediction accuracies of physical and functional protein-protein interaction prediction methods.

KEGG pathway	GN	GC	ES	PP	GM	Average
Amino acid metabolism	0.57	0.52	0.49	0.46	0.49	0.51
Carbohydrate metabolism	0.47	0.50	0.46	0.41	0.41	0.45
Nucleotide metabolism	0.59	0.49	0.44	0.52	0.57	0.52
Lipid metabolism	0.50	0.50	0.45	0.44	0.47	0.47
Metabolism of cofactors & vitamins	0.61	0.53	0.50	0.59	0.57	0.56
Energy metabolism	0.52	0.50	0.51	0.47	0.51	0.50
Metabolism of other amino acids	0.53	0.49	0.48	0.42	0.46	0.48
Glycan biosynthesis & metabolism	0.62	0.58	0.59	0.65	0.56	0.60
Translation	0.76	0.60	0.80	0.79	0.79	0.75
Folding, sorting & degradation	0.68	0.51	0.60	0.72	0.72	0.65
Replication & repair	0.59	0.48	0.50	0.71	0.66	0.59
Signal transduction	0.41	0.48	0.42	0.52	0.42	0.45
Membrane transport	0.39	0.47	0.48	0.41	0.46	0.44
Cell motility	0.62	0.55	0.63	0.66	0.52	0.60
**Average**	**0.56**	**0.51**	**0.53**	**0.56**	**0.54**	

The performance summary of protein-protein prediction methods measured as Area Under the ROC Curve (AUC). Pathway prediction accuracies of various methods differ significantly with Wilcox test p-value <3.582e–13. These methods include Gene Neighbor, Gene Cluster, Expression Similarity, Phylogenetic Profiling and Genome distance-Mirrortree abbreviated as GN, GC, ES, PP and GM respectively. *E. coli* pathways are based on the 2^nd^ level KEGG orthology definition with 50 or more protein components only.

### Prediction of Metabolic Pathway PPIs

**Figure 3:**
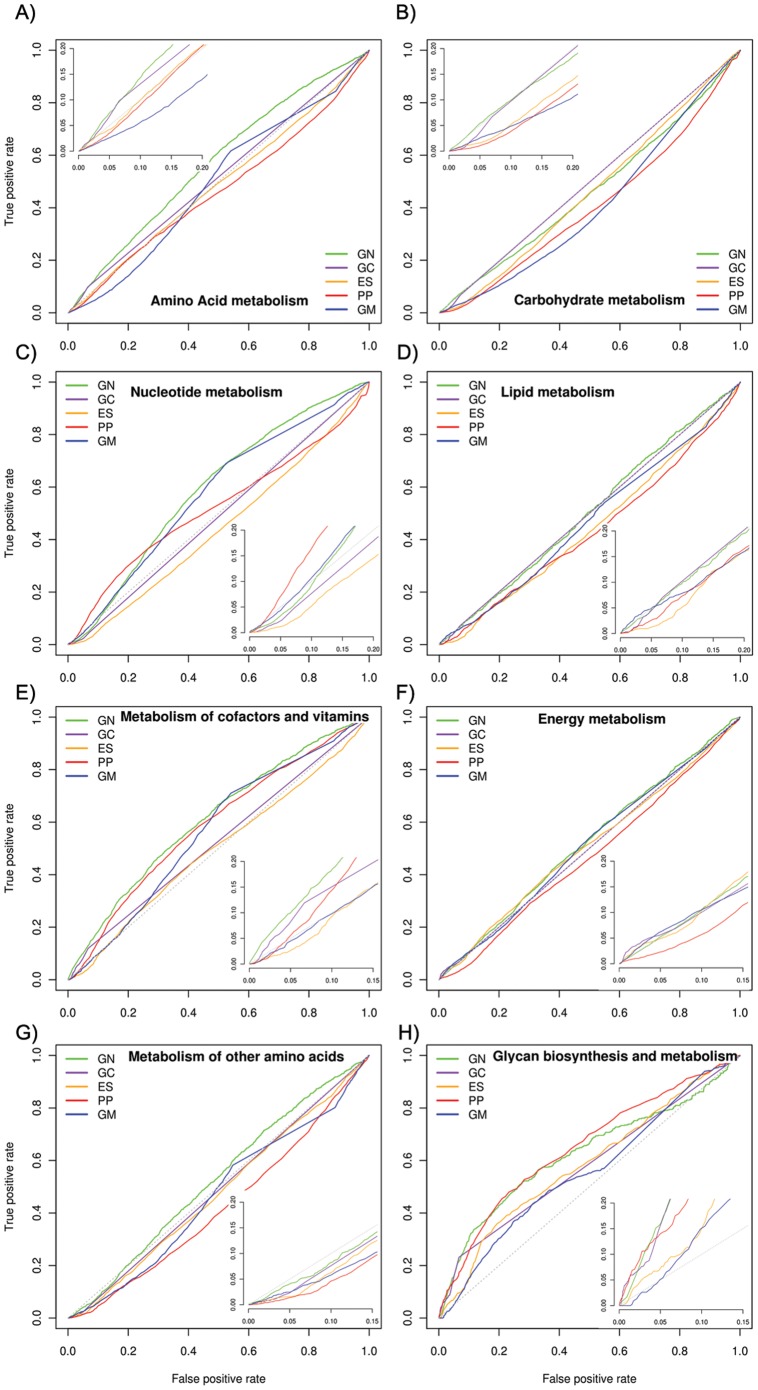
Prediction accuracy of physical and functional protein-protein interactions responsible for metabolism. Each solid colored line represents Receiver Operator Characteristics (ROC) curve of methods. Gray colored dotted line represents performance of random predictor. Gene Neighbor, Gene Cluster, Expression Similarity, Phylogenetic Profiling and Genome Distance-Mirrortree are abbreviated as GN, GC, ES, PP and GM respectively. Each inset on the plot represents performance in the area of high interaction scores generated by prediction methods. Amino acid (A), Nucleotide (C), Co-factors & vitamins (E) and Glycan (H) pathways are predicted with comparable accuracy. Prediction accuracy of Carbohydrate (B), Lipid (D), Energy (F) and Non-standard amino acids (G) pathways by all methods is near random predictor. GN outperforms other methods.

Prediction performance of five methods for detection of functional linkages among proteins belonging to eight metabolic pathways is shown in Figure 3. None of the methods was able to predict linkages among metabolic pathway proteins with high accuracy. Protein associations responsible for metabolism of nucleotide, glycans and vitamins & co-factors were predicted with relatively better accuracy and ROC curves were observed well above random predictor for GN, PP and GC (to some extent) methods (Figure 3C, 3E & 3H). Considering the ability of methods to infer functional relationships between proteins based on evolutionary trajectories, the performance of each method reveals specific evolutionary constraints operational on the pathway protein pairs. GN is performed reasonably well on all these pathway protein linkages followed by poor but noticeable performance of PP (except for amino acid metabolism). These results suggested that proteins belonging to these pathways are encoded by neighboring genes in reference genomes and show weak correlation in their phyletic patterns. Performance of GN also indicated that genes encoding these metabolic proteins tend to be maintained in chromosomal proximity at least in one reference genome and thereby possible co-regulatory nature of these genes (Figure 3). However, the poor performance of ES in predicting linkages among these proteins was intriguing since it suggested that they are not co-regulated (Figure 3 & Table 2). We speculated that it could be due to the alternate transcriptional activity of genes from the same operons or transcriptional units under different conditions as observed for *Mycoplasma pneumoniae* [36]. It has been observed that 42% transcriptional units of *Mycoplasma pneumoniae* show alternate transcript activity [36]. GM method has a potential to detect physically interacting protein pairs as opposed to the other methods which predicts functional PPIs. The poor performance of GM suggested that associations among proteins involved in various pathways (except nucleotide metabolism) are likely to be functional (Figure 3). GM is substantially well predicted nucleotide metabolic protein linkages suggesting that proteins involved in this pathway are associated with each other physically (Figure 3C).

A set of other four pathways responsible for metabolism of carbohydrate, lipid, energy and non-standard amino acids was randomly predicted by all methods (Figure 3B, 3D, 3F & 3G). As shown in Figure 3B, 3D, and 3F, accuracy of PP was worst when it comes to predict functional linkages among proteins involved in carbohydrate, lipid and energy metabolism respectively, suggesting irregular phyletic pattern of these proteins. In case of energy metabolism, our results are consistent with a previous study based on the analysis of bacterial and archaeal key enzymes involved in respiratory and photosynthetic pathways, that the phyletic distribution of these enzymes is irregular and shows diverse strategies adopted by prokaryotes for energy conservation [37]. As opposed to the lower accuracy of PP, functional linkages of carbohydrate, energy and lipid metabolic proteins were predicted comparatively well by GC, suggesting strong preference of genes encoding these proteins being organized as operons during evolution (Figure 3B, 3D, 3F inset). It is also consistent with relative performance of ES in predicting carbohydrate and energy metabolic pathways (Figure 3B & 3F). Nonetheless, predictions of carbohydrate, energy and lipid pathway protein linkages along with the metabolism of non-standard amino acids is a challenging task, since ROC curves of all methods are below random predictor (Figure 3B, 3D, & 3G).

Overall, our results suggest that GN is the best method to predict functional linkages among metabolic proteins. However, as discussed in the previous section (Figure 2), one should treat GN predictions cautiously due to the likelihood of falsely predicted associations even though it is outperformed. The outperformance of GN in predicting linkages among metabolic proteins shed lights on the evolution of pathways. It has been reported in earlier study that gene duplication events have significantly contributed to the evolution of metabolic diversity we observe today [38]. Duplication events often generate new copy of the same gene arranged in-tandem. The fate of the new gene depends on the selection pressure imposed on it and can adopt new but related functions in sub-sequent generations due to the sequence divergence. Our results points out that the organization of extant metabolic genes is shaped by duplication events that arranged same gene in-tandem on the genome during evolution and hence amenable to predicted by GN with high accuracy.

### Prediction of Genetic Information Processing Pathway PPIs

**Figure 4:**
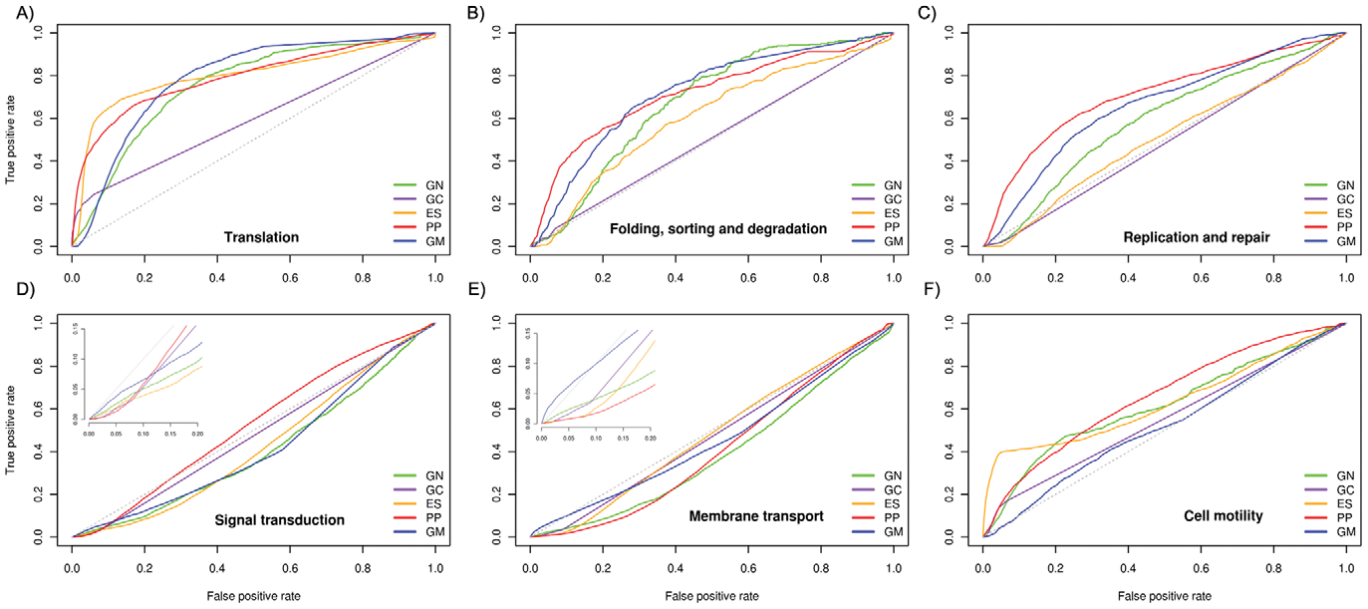
Prediction accuracy of physical and functional protein-protein interactions responsible for various biological pathways. Each solid colored line represents Receiver Operator Characteristics (ROC) curve of prediction methods. Gray colored dotted line represents performance of random predictor. Gene Neighbor, Gene Cluster, Expression Similarity, Phylogenetic Profiling and Genome Distance-Mirrortree are abbreviated as GN, GC, ES, PP and GM respectively. Translation (A), Folding, sorting & degradation (B), and Replication & repair (C) are well predicted by PP and GM. Signal transduction (D) and Membrane Transport (E) pathways are predicted randomly by all PPI prediction methods. GM performed well as compared to other methods in low false positive region (D & E Inset). PP, ES and GN elegantly predicted interactions among proteins involved in Cell motility pathway (F).

Central dogma of life related pathways with 50 or more protein components namely, translation, protein folding, sorting & degradation and replication & repair pathways are classified under genetic information processing term in KEGG (Figure 4A, 4B and 4C). Translation pathway PPIs were exceptionally well predicted by methods other than GC (Figure 4A). PP and ES methods showed remarkable ROC curves at low FP rate whereas GM and GN methods for full range of values (Figure 4A). The top scoring AUC values of 0.80, 0.79 and 0.79 were observed for ES, PP and GM method respectively. PP and ES methods predicted more than 50% TP at the cost of less than 10% FP. Similar percentage of TP was obtained for GM and GN at the cost of higher FP. These results implied that the predicted associations are physical, co-inherited and co-regulated which was expected since these proteins form macromolecular assemblage of ribosome crucial for synthesis of proteins and are expected to be constitutively present in the cell associated with each other.

Another housekeeping function performed during the protein synthesis is proper folding of nascent polypeptide followed by its transport to respective locations in the cell and degradation, if not folded properly or when their function is no longer needed. The best performing methods to predict folding, sorting and degradation pathways were PP and GM suggesting co-inheritance of the protein components and their physical association with each other (Figure 4B). The AUC value of 0.72 was observed for PP and GM methods. Overall PP, GM and GN methods are best suited for prediction of protein associations involved in this category (Table 2). The poor performance was observed for GC, suggesting that these protein coding genes are not organized as operons, which was opposed to the performance of GN reflecting chromosomal proximity of these protein coding genes during evolution.

Replication and repair pathway protein components are involved in maintenance of chromosomes. This also includes one of the indispensable functions, the replication of DNA molecules during cell division which is common to life forms. The linkages among these proteins were mainly predicted by PP with AUC value of 0.71 followed by GM with 0.66. ROC curves are well separated and above right diagonal suggesting that the predictions could be achieved with reasonable accuracy (Figure 4C). These results suggested that these proteins are co-inherited during evolution and interact physically.

Overall, genetic information processing pathway proteins were maintained by strong co-inheritance patterns and they are possibly associated with each other by physical interactions in the cell.

### Prediction of Environmental Information Processing Pathway PPIs

Signal transduction and membrane transport pathways were predicted with poor accuracy (Figure 4D and 4E). The interactions involved in signal transduction proteins were mainly predicted by PP method with AUC value of 0.52 (Figure 4D). The second best performance was observed in the case of GC method with AUC value of 0.48. Membrane transport protein linkages were also predicted randomly (Figure 4E). The performance of all methods was observed well below the random predictor. The best AUC values of 0.48 and 0.47 were achieved for ES and GC methods respectively. In the low FPR region, the performance of GM was slightly better compared to other methods for both pathways suggesting some of these pathway proteins may form complexes through physical interactions (Figure 4 Inset D and E).

Overall our results suggested that the prediction of signaling and membrane transport pathway PPIs is a daunting task for all methods.

### Cellular Processes

Cell motility related proteins are categorized under the term ‘Cellular processes’ in the KEGG database and are sub-divided into chemotaxis and flagellar assembly pathways. These PPIs were predicted relatively better by PP and ES methods with AUC values of 0.66 and 0.63 respectively (Figure 4F). The outperformance of PP was expected since the proteins responsible for motility are restricted to motile organisms and show very strong co-inheritance pattern. The second best performance was observed for ES suggesting co-expression of these proteins. The lowest performance with 0.42 AUC was observed for the GM. Sub-cellular localization of these proteins suggests that many proteins actually not interacting with each other but associated by adjacent neighbors into complex [39]. Furthermore, chemotaxis proteins are involved in signaling more often bound by transient interaction to induce phosphorylation events which could be the reasons for poor performance of GM [40].

### Conclusions

The comparative assessment of five methods for their ability to predict protein-protein functional linkages of various biological pathways showed that every method is prone to erroneous predictions. The prediction of associations among the metabolic pathway proteins remains a challenging task for these methods, possibly due to their flexible/independent evolutionary histories. The lower performance of PP method has indicated an irregular phylogenetic distribution of proteins involved in the majority of metabolic pathways. These metabolic pathways are responsible for the turnover of carbohydrates, amino acids, lipids and non-standard amino acids. The PP method delineates the fact that, 1) the most of the metabolic genes are dispensable due to functional redundancy and this could be the underlying reason for weak correlation in their phyletic patterns, 2) Besides, each metabolic pathway has evolved around few core genes that are well conserved, whereas majority of other genes are versatile enough to offer a species/ecological niche specific framework for the environmental adaptation. Since, the proteins involved in metabolism of nucleotides, glycans, vitamins and co-factors are well conserved, PP and GN methods worked with greater accuracy.

Majority of metabolic pathway protein linkages ranked well by gene neighborhood based method suggesting that the genes encoding them are in close proximity to each other on chromosome in one of the reference genomes. Such organization of genes involved in the extant metabolic pathways are probably shaped and arisen due to duplication of the same genes in-tandem. GN and GC methods could be used as powerful tools to predict functional linkages among the metabolic proteins. On other hand, GM method could make reliable predictions as far as nucleotide metabolic protein-protein linkages are concerned.

As opposed to the above-mentioned lower performance in predicting metabolic protein linkages, these methods are extremely good in detecting interactions among proteins that are responsible for housekeeping processes and other subset of processes such as cell motility. Functional linkages between proteins that participate in the central dogma pathway can be predicted with reliable accuracy by PP, GM and ES methods. Higher prediction accuracies for these pathways were obvious since participating proteins form small complexes or molecular machines that are essential for genetic information processing. However, we suggest that these proteins not only show strong co-inheritance but also interact physically being co-expressed. Prediction of information processing pathways such as signal transduction and membrane transport remains the challenging task for these methods. None of the methods could show enhanced accuracy than the random predictor in detecting associations among these proteins.

Metabolic and information processing pathway predictions with respect to the central dogma, consistently showed very small AUC values. These AUC values ranged mostly from 0.5 to 0.6 and can be rated slightly above random performance, indicating high proportion of false positive predictions. The acceptable range of AUC values from these methods have been achieved only for the central dogma pathways. Therefore, we believe the reported accuracies in all previous prediction analyses are likely to be the overestimated and resulted mainly from central dogma pathway predictions. These facts suggest that majority of methods used in this study are not enough to predict metabolic and information processing pathways with enough accuracy. Furthermore, the genome-scale predictions made by these methods are likely to have less coverage of protein linkages involved in these pathways. Furthermore, the careful selection of prediction methods along with pathway specific sequence features would enhance the prediction quality.

In concluding remarks, overall AUC values of predictions are not very convincing for majority of the pathways. There is a differential tendency of various methods to predict various biological pathways. Therefore, we suggest that the potential use of a particular prediction method depends on the pathway under investigation. In making generalized prediction, ES method is the best option.

## Materials and Methods

Completely sequenced genomes of Bacteria available as on December 2007 at National Center for Biotechnology Information (NCBI) were downloaded from ftp://ftp.ncbi.nih.gov/genomes/Bacteria [41]. A total of 566 prokaryotic species with single chromosomes were considered for analysis. We selected *Escherichia coli* K12 MG1655 (*E. coli*) as a query/model organism. Orthologs of *E. coli* proteins were identified by reciprocal best hit search performed using NCBI Basic Local Alignment Search Tool (BLAST) [42] against remaining 565 genomes. The hits with e-value threshold of 1e-4 or less and bits score greater than 50 were retained as potential orthologs.

### PPI Prediction Methods

Interaction scores for all possible pairs of *E. coli* proteins were calculated using five PPI prediction methods which include GN, GC, ES, PP, and GM methods. In our previous study, we benchmarked methods other than ES against six reference genome sets (Text S1 ) [34]. Given two proteins X and Y of the query genome, these methods generate interaction score based on various aspects of their evolution computed through orthologs in a set of reference genomes. A set with 121 reference genomes representing single species from various genus and related genera was performed relatively better when GN, GC and GM methods were used for predictions [34]. Therefore, we used these genomes as a reference set to compute GN, GC and GM interaction scores for gold standard protein pairs (discussed in the next section).

Briefly, GN method computes interaction score for a protein pair based on the chromosomal distance between genes encoding their orthologs from any one reference genome in which it is found minimum [21]. We subtracted each GN score from one to make it similarity score instead of distance. GC method computes co-occurrence probability for *E. coli* protein pair based on the coding genes of orthologous proteins in the same gene clusters of reference genomes [21]. The gene clusters defined in all reference genomes, as a continuous stretches of co-directional genes with intergenic distance cutoff within 100 nucleotide bases.

GM or Genome distance-Mirrortree method is a variant Tol-mirrortree [25], [34]. For GM, Orthologs of *E. coli* proteins were identified in 121 reference genomes. Orthologs of each protein were used to construct Multiple Sequence Alignments (MSAs) [43]. MSAs were used to calculate distance matrices for each protein of *E. coli.* The dimension of the each protein distance matrix would be equal to the number of genomes/species in which orthologs of that protein identified. Suppose, protein ‘X’ is identified in *n* species then the dimension of the matrix *D*X would be *n* x *n*. Each row or column of the matrix corresponds to a species under consideration. An element of the matrix *D*X (*i,j*) represents the distance calculated by comparing the amino acid sequences of orthologs of protein X from species *i*, and *j*. Similarly, distance matrix derived for protein ‘Y’ using MSA of its orthologs from *n* species. To correlate the distance matrices of protein X and Y, the distance between the species that were common to both matrices were retained. All the distance matrices were symmetric, hence upper or lower half of the matrix can be used for comparison. Since the distances that correspond to common species between two matrices were retained, one can use standard Pearson Correlation Coefficient (PCC) to calculate the extent to which two distance matrices are similar. The higher PCC value suggests the correlated amino acid substitutions in the orthologs of protein X and Y. Hence, it is likely that protein X and Y interact physically. There are several ways to exclude the speciation information or background noise due to the relatedness of species from these matrices [25], [44]. In our previous study [34], we have used novel approach which is similar to Tol-mirrortree to correct these matrices for speciation bias [25]. For 121 reference genomes, we first created distance matrix *D*G which is similar to protein matrices where each row or column corresponds to a species/genome under consideration. An element of the matrix *D*G (*i,j*) represents the Genome Distance (GD) based on shared orthologs between species *i* and *j* which was calculated with the following equation,
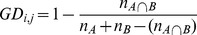
Where, *n_A_* and *n_B_* is the total number of proteins present in species *i* and *j*, respectively. *n_A∩B_* is the number of orthologs shared by species *i* and *j*. The orthologs were obtained for each of the species using a bi-directional BLAST searches against each other.

Each protein distance matrix was corrected by subtracting its elements from the corresponding value in the *D*G matrix (i.e. distance value of an element (*i,j*) of protein X was subtracted from the (*i,j*) element of matrix *D*G). Prior to correction, all matrices were re-scaled due to the different scale of proteins and GD matrix. For this, we calculated PCC between *D*G matrix and every distance matrix of *E. coli* proteins. The highest PCC value obtained was 0.54 which was used to divide elements of each matrix before subtraction. This approach is referred as GD-Mirrortree (GM) and performed better than tol-mirrortree method [34].

A set of 448 reference genomes was created by selecting one genome from a cluster obtained using the genomes that share more than 90% of *E. coli* orthologs [34]. This reference set was used for PP analysis. Phylogenetic profile matrix (P) was created where each row and column of matrix represent *E. coli* protein and reference genome respectively [15], [45]. An P(*i,j*) element of the matrix represents bit score of alignment between protein *i* and its ortholog in reference genome *j*. Matrix was normalized with respect to protein and species divergence [21]. The similarity of phylogenetic profiles was assessed using PCC.

Similarly, the gene expression data for 380 conditions was downloaded from M3D database [46]. The most varying 300 conditions were used to calculate the expression similarity of *E. coli* protein coding gene pairs using PCC.

### Performance Measures

To assess the performance of each PPI prediction method with respect to KEGG pathways, protein pair was considered as functionally linked or positive if both proteins shared at least one KEGG pathway, and unrelated otherwise i.e. negative. For analysis of individual KEGG pathways, however, two proteins were considered to be functionally linked or positive if they belonged to the same KEGG pathway, otherwise unrelated or negative with respect to pathway under consideration [28], [29]. For individual pathway analysis, the gold standard protein pairs share at least one functional pathway or category.

For a chosen threshold interaction score calculated by PPI prediction methods, the protein pairs with scores greater than or equal to the threshold and belong to positive examples are classified as True Positives (TP) and those with a interaction scores below the threshold are classified as False Negatives (FN). Similarly, the protein pairs with interaction scores greater than or equal to the threshold and belong to negative examples are classified as False Positives (FP) and those with a score below the threshold are classified as True Negatives (TN).

These labels were used to assess the performance of PPI prediction methods in the form of TP to FP ratios. ROC curves and AUC values were calculated using ROCR package in R language for statistical programming (http://www.r-project.org/) [47]. For ROC analysis, True Positive Rate (TPR) and False Positive Rate (FPR) are as following
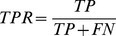


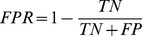



ROC curve visually represents the relative trade-offs between the FPR and the TPR. A correct PPI prediction method would have a ROC curve above diagonal and its integral the AUC would be above 0.5. For 100% correct predictions, this curve is rectangular and AUC is equal to 1.

## Supporting Information

Table S1 **Description of the computational cost for analyses.**
(PDF)

Text S1 **Benchmarking details of physical and functional protein-protein interaction prediction methods.**
(PDF)
