# Supplementary material for: Evaluation of Physical and Functional Protein-Protein Interaction Prediction Methods for Detecting Biological Pathways
Source: PLoS One. 2013 Jan 17;8(1):e54325. doi: 10.1371/journal.pone.0054325 (PMC3547882; doi:10.1371/journal.pone.0054325)
Supplement: Table S1 — Description of the computational cost for analyses. (PDF) [file pone.0054325.s001.pdf]

Step No.	Description of job/step	Execution time (hours: minutes)	Remark/Details
**1**	Bidirectional BLAST search of E. coli proteins against database of 565 organisms	12:47	4132 E. coli proteins were searched against 1,816,355 protein sequences. The best bidirectional BLAST hits were detected for 4038 out of 4132 proteins at least in one organism with mean value of 145 organisms.
**2**	Bidirectional BLAST search of protein sequences from each of 123 genomes against remaining genomes	118:00	This dataset was used to calculate distance between two genomes based on shared homologs. These distances were used to correct phylogenetic distance matrices constructed for GM analyses
**3**	Multiple Sequence Alignment (MSA) construction for GM analyses.	11:56	This step involves MSA construction for 2628 E. coli proteins for which we could detect homologs in at least 10 out of 123 reference organisms.
**4**	Phylogenetic profiling calculations (PP)	00:42	Phylogenetic profiles constructed for 1,393 against 448 organisms. Each profile then compared with remaining using PCC
**5**	Gene Expression Similarity (ES)	00.26	Expression profile of each protein coding gene in 300 conditions was compared with remaining profiles using PCC
**6**	Genome-distance Mirrortree Method (GM)	12:24	Distance matrices of 1,393 proteins constituting 969,528 pairs compared using PCC. MSAs constructed in step 3 were used for the creation of distance matrices
**7**	Gene Cluster Method (GC)	00:04	123 reference organisms used for analysis. It takes less than 2 minutes to define gene clusters in reference genomes based on intergenic distance criteria.
**8**	Gene Neighbour Method (GN)	00:06	123 reference genomes used for analysis
**9**	TP, FP, FN, and TN calculations at 0.02 interval of scores generated by each method for 969,528 protein pairs (The scores ranged between 0 to 1)	00:12	For PP method
		00:13	For ES method
		00:12	For GC method
		00:13	For GM method
		00:12	For GN method

Notes: The total number of hours and minutes for each step calculated based on start and end time/date of the job. Above mentioned analyses performed on 64-bit HP xw9000 workstation with 8 CPUs and 8 GB RAM.
