# Supplementary material for: Evaluation of Physical and Functional Protein-Protein Interaction Prediction Methods for Detecting Biological Pathways
Source: PLoS One. 2013 Jan 17;8(1):e54325. doi: 10.1371/journal.pone.0054325 (PMC3547882; doi:10.1371/journal.pone.0054325)
Supplement: Text S1 — Benchmarking details of physical and functional protein-protein interaction prediction methods. (PDF) [file pone.0054325.s002.pdf]

Benchmarking details of physical and functional protein-protein interaction prediction methods

GN, GC, PP and GM methods require a set of reference genomes for analysis. Given two proteins X and Y of the query genome, each prediction method generates a numerical value (or interaction score) based on various aspects of their evolution computed through orthologs in a set of reference genomes. Therefore, each prediction method requires a set of reference genomes to compute interaction scores for protein pairs. In our previous study [1], we created six sets of reference genomes from initial set of 565 that are called ALL, BAAC, BAS, BAC, GAMMA and BANR. The total number of genomes in "ALL" set included all the 565 prokaryotic genomes. BAAC set was composed of 448 diverse reference genomes, automatically detected based on the shared orthologs of *E. coli* proteins between them. To remove reference genomes with similar proportion of orthologs detected in *E. coli,* a fraction of similarity between the two reference genomes was calculated using Tanimoto coefficient [1]. The resulting Tanimoto coefficients (S_{AB}) between all possible pairs of reference genomes were sorted and those having coefficient of 0.9 or more were selected for clustering. The clustering was carried out using Markov Cluster algorithm (MCL) [2] and only one genome was retained from each cluster. A total of 448 genomes remained after filtering 117 out of 565 original reference genomes. "BAS" set included 121 phylogenetically diverse genomes representing single genomes from various genera. "BAC" set exclusively represented genomes of 86 phylogenetically diverse eubacterial species. Similarly, we created "GAMMA" set represented by 46 γ-proteobacterial genomes and " BANR" set represented by 41 reference genomes including 20 bacteria and 21 archaea. This filtering step was used to minimize the overrepresentation of certain genomes as many genera of prokaryotes have single species while others have multiple species.

We observed that the BAC reference set of 121 phylogenetically diverse genomes performed relatively well when GN, GC and GM methods were used to detect physical and functional protein-protein associations reported in DIP, EcoCyc and KEGG databases [1]. Likewise, PP method outperformed when BAAC reference set of 448 genomes was used to construct phylogenetic profiles. Therefore, in the present study we have used BAS set of

121 reference genomes to predict functional linkages by using GN, GC and GM methods, whereas for PP we have used BAAC set of 448 reference genomes.

The methods other than GM and ES (these are explained in main text) were implemented as below [1],

Phylogenetic Profiling Method

Phylogenetic profile matrices were created for BAAC reference genome set. Rows in matrix were *E. coli* proteins, $i_1, i_2...i_{4132}$ and columns were reference genomes, $j_1, j_2...j_n$, where n is the number of genomes in a reference set i.e. 448. Each (i,j) cell of this matrix was filled with the bit score of *E. coli* protein i and its homolog in the jth reference genome. If a protein was absent in any reference genome then it was denoted with score zero. Each cell or point of the phylogenetic profile matrix of a protein, i (i.e., row) was normalized as $NBS_{ij} = BS_{ij}/BS_{max}$, where BS_{ij} is the bit score of the alignment between *E. coli* protein i and its ortholog in reference genome j. BS_{max} is the maximum value of bit score obtained for protein i over all its orthologs from n reference genomes. Second normalization was carried out on reference genomes (i.e., column) by dividing the minimum bit score over all *E. coli* protein orthologs in j^{th} reference genome. Two proteins X and Y of *E. coli* displaying similar phylogenetic profiles were assessed by calculating standard Pearson Correlation Coefficient (PCC) between their vectors.

Gene Cluster Method (GCM)

A gene cluster in a genome is defined as a set of continuous co-directional genes with an intergenic distance of 100 nucleotide bases or less between them. The gene clusters were identified in 121 reference genomes (i.e, BAS set). The propensity scores for KEGG protein pairs were calculated as,

$$propensity_{(X,Y)} = \frac{1}{n}\sum_{i=1}^{n} XY$$

Where n is the number of genomes in a reference set, XY=1 if orthologs of *E. coli* protein X,Y $\in$ gene cluster in i^{th} reference genome, otherwise 0.

Minimum Distance Method (MDM)

The minimum distance between genes encoding protein X and Y of *E. coli* on the basis of genes encoding their orthologs in the reference genomes is calculated. Briefly, if the query proteins X and Y are present in the reference genome *i*, then the probability that genes encoding their orthologs are separated by fewer than *d* nucleotide bases is given by

$$p_{i=1}^{n}(\leq d) = \frac{2d}{N}$$

Where, *d* is the distance between translation start sites of genes encoding orthologs of X and Y in the ith reference genome. *N* is the length of the chromosome of ith reference genome in nucleotide bases. *n* is the total number of reference genomes in a set. Since the genomes under consideration are circular, the distances between the gene pairs were calculated in both clockwise and anti-clockwise direction. Minimum of these two values is *d*. The minimum probability in any one reference genome is considered as the interaction score for query proteins X and Y.

References

1. Muley VY, Ranjan A (2012) Effect of reference genome selection on the performance of computational methods for genome-wide protein-protein interaction prediction. PLoS One 7: e42057.
2. Van Dongen S (2000) Graph clustering by flow simulation [PhD thesis]. Utrecht: Universityof Utrecht.
